# WRKY transcription factors modulate flowering time in four *Arachis* species: a bioinformatics analysis

**DOI:** 10.1186/s12870-024-05343-7

**Published:** 2024-06-28

**Authors:** Xiao Fang, Lubin Liu, Meiran Li, Hui Song, Yihui Zhou

**Affiliations:** 1 School of Animation and Media, Qingdao Agricultural University, 700# Changcheng Road, Qingdao, Shandong 266019 China; 2 College of Grassland Science, Qingdao Agricultural University, 700# Changcheng Road, Qingdao, Shandong 266019 China

**Keywords:** *Arachis* genus, Flowering time, Protein interaction, WRKY transcription factor

## Abstract

**Background:**

WRKY proteins are important transcription factors (TFs) in plants, involved in growth and development and responses to environmental changes. Although WRKY TFs have been studied at the genome level in *Arachis* genus, including oil crop and turfgrass, their regulatory networks in controlling flowering time remain unclear. The aim of this study was to predict the molecular mechanisms of WRKY TFs regulation flowering time in *Arachis* genus at the genome level using bioinformatics approaches.

**Results:**

The flowering-time genes of *Arachis* genus were retrieved from the flowering-time gene database. The regulatory networks between WRKY TFs and downstream genes in *Arachis* genus were predicted using bioinformatics tools. The results showed that WRKY TFs were involved in aging, autonomous, circadian clock, hormone, photoperiod, sugar, temperature, and vernalization pathways to modulate flowering time in *Arachis duranensis*, *Arachis ipaensis*, *Arachis monticola*, and *Arachis hypogaea* cv. Tifrunner. The WRKY TF binding sites in homologous flowering-time genes exhibited asymmetric evolutionary pattern, indicating that the WRKY TFs interact with other transcription factors to modulate flowering time in the four *Arachis* species. Protein interaction network analysis showed that WRKY TFs interacted with FRUITFULL and APETALA2 to modulate flowering time in the four *Arachis* species. WRKY TFs implicated in regulating flowering time had low expression levels, whereas their interaction proteins had varying expression patterns in 22 tissues of *A. hypogaea* cv. Tifrunner. These results indicate that WRKY TFs exhibit antagonistic or synergistic interactions with the associated proteins.

**Conclusions:**

This study reveals complex regulatory networks through which WRKY TFs modulate flowering time in the four *Arachis* species using bioinformatics approaches.

**Supplementary Information:**

The online version contains supplementary material available at 10.1186/s12870-024-05343-7.

## Background

WRKY transcription factors (TFs) modulate plant growth and development and response to abiotic and biotic stress by regulating downstream genes and forming protein complexes [1–5]. WRKY TFs modulate the plant flowering process through various pathways, including photoperiod, autonomous, vernalization, gibberellin, and aging pathways [4, 6]. AtWRKY71 from *Arabidopsis thaliana* activates *FLOWERING LOCUS T* (*FT*) and *LEAFY* (*LFY*) to promote flowering [7]. AtWRKY75 interacts with DELLA to activate *FT*, accelerating flowering [8]. Moreover, WRKY184 from *Brassica napus* upregulates *FRUITFULL* (*FUL*) expression to promote flowering [9]. WRKY TFs are also implicated in phytohormone pathways, such as abscisic acid (ABA) and auxin, to modulate flowering time. OsWRKY72 from *Oryza sativa* indirectly activates genes associated with the auxin pathway, such as *AUXIN1*, *AUXIN RESISTANT1*, and *BUD1*, and genes related to the ABA pathway, such as *ABA2* and *ABA INSENSITIVE 4* (*ABI4*), to promote flowering [10]. WRKY TFs regulate salt and cadmium stress to modulate flowering time. For instance, RtWRKY23 from *Reaumuria trigyna* upregulates *HISTONE ACETYLTRANSFERASE 1*, *ANT*, and *MADS AFFECTING FLOWERING 5* expression to alleviate salt stress, resulting in earlier flowering [11]. AtWRKY12 and AtWRKY13 potentially co-regulate flowering time and cadmium stress due to their similar regulatory patterns in modulating flowering time and cadmium stress [4].

Environmental changes, such as nutrient availability, drought stress, and temperature stress, can alter flowering time [4]. However, the role of WRKY TFs in regulating these pathways ultimately modulating flowering time is unclear. This indicates that WRKY TFs may regulate flowering time through additional flowering pathways. Therefore, it is imperative to conduct research to identify flowering-time genes at the genome level and verify whether WRKY TFs regulate these genes. A plant flowering-time gene database, PFGD, was established in the recent past [12]. The database provides a platform to identify WRKY TFs that regulate downstream genes and the proteins associated with these TFs.

Plants in the *Arachis* genus serve as oil, forage and turfgrass crops [13]. Cultivated peanut (*Arachis hypogaea*) and *Arachis monticola* are allotetraploids obtained by crossing by two wild diploids, *Arachis duranensis* and *Arachis ipaensis* [14, 15]. Comparative genomic analyses between diploid and tetraploid peanuts reveal asymmetric evolution in the subgenomes of cultivated peanuts [16–18]. Asymmetric evolution of subgenomes leads to functional bias of genes in a specific subgenome [16, 17, 19]. A homoeologous WRKY pair, AhTWRKY24 from subgenome B and AhtWRKY106 from subgenome A, were identified in *Arachis hypogaea* cv. Tifrunner [20]. DNA affinity purification sequencing data revealed that AhTWRKY24 and AhtWRKY106 regulate approximately an equal number of downstream genes in *A. hypogaea* cv. Tifrunner genome, but they also exhibit specific regulation of distinct downstream genes [20]. These results indicate that asymmetric evolution influences genes regulated by WRKY in *A. hypogaea* cv. Tifrunner.

Although WRKY TFs have been identified at the genome level in members of *Arachis* genus [5, 21], their role in regulating flowering time is yet to be fully elucidated. In this study, flowering-time genes for *A. duranensis*, *A. ipaensis*, *A. monticola*, and *A. hypogaea* cv. Tifrunner were retrieved from PFGD database. This study revealed the regulatory networks of WRKY TFs in the four *Arachis* species through bioinformatics approaches.

## Methods

### Sequence retrieval

The PFGD database (http://pfgd.bio2db.com/index.html) is a valuable repository for genes that regulate flowering time in plants [12]. The flowering-time genes of the *Arachis* species were retrieved from the PFGD database. The species of the *Arachis* genus included *A. duranensis*, *A. ipaensis*, *A. monticola*, and *A. hypogaea* cv. Tifrunner.

WRKY TFs have been identified in various *Arachis* species [5, 21]. *A. duranensis*, *A. ipaensis*, *A. monticola*, and *A. hypogaea* cv. Tifrunner have 16 WRKY TFs, which are AtWRKY12 and AtWRKY75 homologs and are involved in regulating flowering time [5, 21, 22]. The 16 WRKY TFs in the four *Arachis* species were retrieved from PeanutBase database based on previous studies [5, 21, 22].

The orthologs of the genes in *A. duranensis*, *A. ipaensis*, *A. monticola*, and *A. hypogaea* cv. Tifrunner were identified using the MCScan X program with an e-value of 1E-10 [23]. Similarly, paralogs and homoeologs were identified using the MCScan X program. The homoeologs were identified in *A. monticola* and *A. hypogaea* cv. Tifrunner.

### Identification of WRKY TFs regulation flowering-time genes in *Arachis* genus

The 2 kb *cis*-acting regions of flowering-time genes were isolated from the four *Arachis* species and uploaded to the Nesite database to predict the WRKY TF binding sites (TFBSs) [24]. The search parameters were an expected mean number of 0.01, a statistical significance level of 0.95, an 80% homology between known TFBS and motif, and a 20% variation in the distance between TFBS blocks. The protein interaction relationships between the 16 WRKY TFs and flowering-time proteins in the four *Arachis* species were predicted using the STRING database. *A. thaliana* was used as a reference and the protein–protein interaction network analysis was conducted with the default parameters in the STRING database.

### Tissue expression profile of *WRKY* and flowering-time genes in* A. hypogaea* cv. Tifrunner

The RNA-seq datasets of 22 tissues of *A. hypogaea* cv. Tifrunner were retrieved from PeanutBase [25, 26]. The raw read counts were aligned to the *A. hypogaea* cv. Tifrunner genome using Bowtie 2 in the TBtools program [23], and expression levels were quantified as fragments per kilobase of transcript per million mapped reads (FPKM) using RSEM [27]. The expression levels were standardized by log_2_ transformation (FPKM + 1). The expression patterns were visualized using TBtools program [23].

### Phylogenetic analysis

MAFFT was used to conduct multiple sequence alignments with default parameters [28]. ProtTest was used to estimate the best-fit model for the construction of a phylogenetic tree based on the maximum likelihood method [29]. The IQ-tree program was used to construct the maximum likelihood trees using the best-fit model from the ProtTest program, 10,000 ultrafast bootstraps, and 1000 the SH-like approximate likelihood ratio test. The phylogenetic trees were visualized using the Figtree tool [30].

## Results

### Multiple flowering-time pathways are identified in *Arachis* genus

**Fig. 1:**
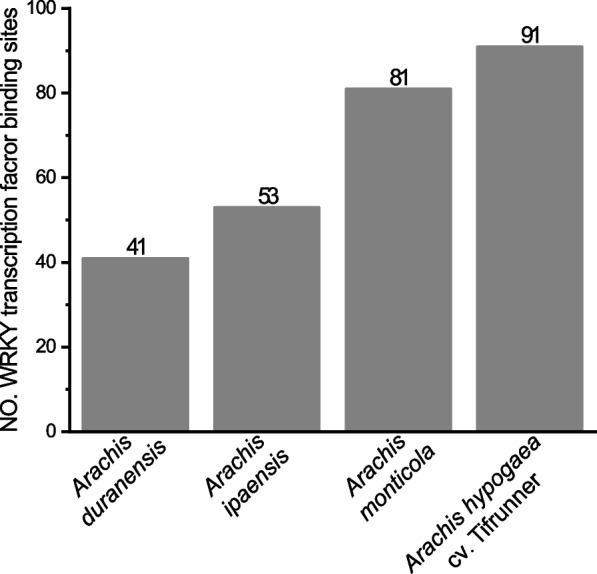
The flowering-time genes regulated by WRKY transcription factors in the four *Arachis* species

The PFGD database comprises 552, 622, 514, and 576 flowering-time genes from *A. duranensis*, *A. ipaensis*, *A. monticola*, and *A. hypogaea* cv. Tifrunner (Accessed on January 23, 2024). Notably, analysis of the *cis*-acting elements showed that 41, 53, 81, and 91 flowering-time genes are potentially regulated by WRKY TFs in *A. duranensis*, *A. ipaensis*, *A. monticola*, and *A. hypogaea* cv. Tifrunner (Fig. 1 and Additional File 1). These four species of *Arachis* genus had similar flowering pathways regulated by WRKY TFs. The flowering pathways included aging, autonomous, circadian clock, hormone, photoperiod, sugar, temperature, and vernalization. However, no  previous evidence confirms the involvement of WRKY TFs in sugar and temperature pathways to modulate flowering time.

### Homologous flowering-time genes have asymmetric WRKY transcription factor binding sites

**Fig. 2:**
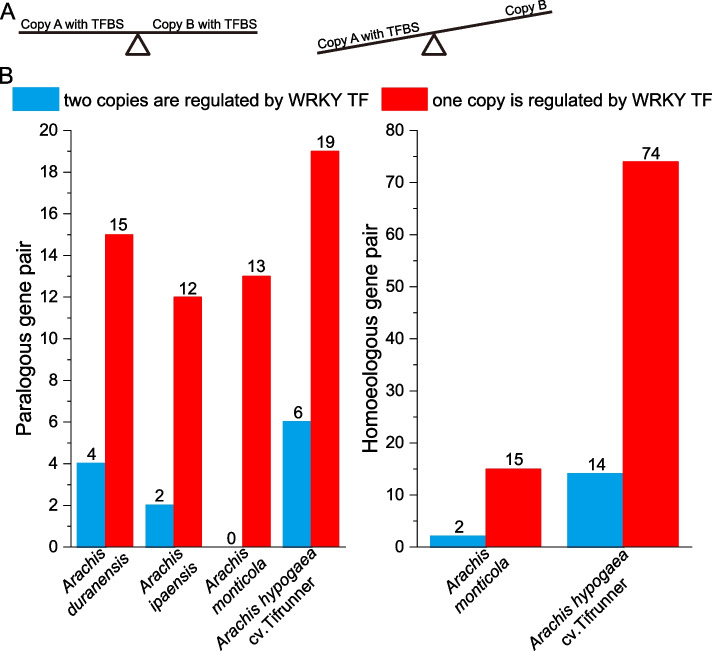
Homologs potentially regulated by WRKY transcription factors. **a** A schematic representation of symmetric and asymmetric WRKY transcription factor binding site (TFBS) in a homologous pair. **b** The homologs of flowering-time genes exhibiting WRKY TFBSs in the four *Arachis* species

The four species in *Arachis* genus have different numbers of flowering-time paralogs. In *A. duranensis*, four pairs of paralogous flowering-time genes contained WRKYTFBSs, whereas 15 flowering-time genes from 15 paralogous pairs contained WRKY TFBSs (Fig. 2 and Additional File 2). Similarly, *A. ipaensis* and *A. hypogaea* cv. Tifrunner had two and six pairs of paralogous flowering-time genes containing WRKY TFBSs, respectively (Fig. 2 and Additional File 2). Moreover, one copy of 12, 13, and 19 paralogous flowering-time gene pairs in *A. ipaensis*, *A. monticola*, and *A. hypogaea* cv. Tifrunner exhibited WRKY TFBSs (Fig. 2 and Additional File 2). These results indicate that evolutionary patterns of WRKY TFBSs in flowering-time genes vary across species.

The findings showed that *A. monticola* and *A. hypogaea* cv. Tifrunner have varying number of homoeologs associated with flowering time. *A. monticola* and *A. hypogaea* cv. Tifrunner exhibited two and 14 homoeologous flowering-time gene pairs containing WRKY TFBSs, respectively. Conversely, *A. monticola* and *A. hypogaea* cv. Tifrunner had one copy of 15 and 74 homoeologous flowering-time gene pairs exhibiting WRKY TFBSs (Fig. 2 and Additional File 2). These results indicate the WRKY TFBSs in flowering-time genes exhibit an asymmetric evolutionary pattern between the two species.

Previous studies demonstrated that 16 *Arachis* WRKY TFs are orthologs with AtWRKY12 and AtWRKY75, which regulate flowering time by binding W-box elements of *FUL* and *FT* genes [8, 31]. Conserved orthologs of flowering time were identified across four *Arachis* species through synteny analyses. These genes are mainly implicated in aging, autonomous, and sugar pathways (Table 1). These results indicate that WRKY TFs modulate specific regulatory networks of flowering time in the four *Arachis* species.

**Table 1:** WRKY transcription factors potentially regulate conserved orthologs in the four *Arachis* species

duranensis	ipaensis	monticola	Tifrunner	Genome	Arabidopsis	Annotion	Pathway
Aradu.EI58K	No component	EVM0038983	T5C9EA	A	AT5G44160	INDETERMINATE DOMAIN 8, NUTCRACKER	Sugar
No component	Araip.6UE4Z	Not determined	V8NH83	B			
Aradu.F7PQZ	No component	Not determined	Not determined	A	AT1G78580	TREHALOSE-6-PHOSPHATE SYNTHASE 1	Aging
No component	Araip.FER8B	EVM0016112	PK6QLT	B			
Aradu.FY71B	No component	Not determined	8JQ024	A	AT3G11910	UBIQUITIN-SPECIFIC PROTEASE 13	Autonomous
No component	Araip.QTU2G	EVM0050491	Not determined	B			
Aradu.NJJ5I	No component	Not determined	Q6VMSA	A	AT3G63010	GA INSENSITIVE DWARF 1B	Aging
No component	Araip.6D3E7	EVM0030129	Not determined	B			
Aradu.SE6Q0	No component	EVM0035381	00XS3D	A	AT5G67180	TARGET OF EARLY ACTIVATION TAGGED 3	Aging
No component	Araip.MAL00	Not determined	Not determined	B			

### WRKY TFs interact with FUL and AP2 to modulate flowering time in *Arachis* genus

**Fig. 3:**
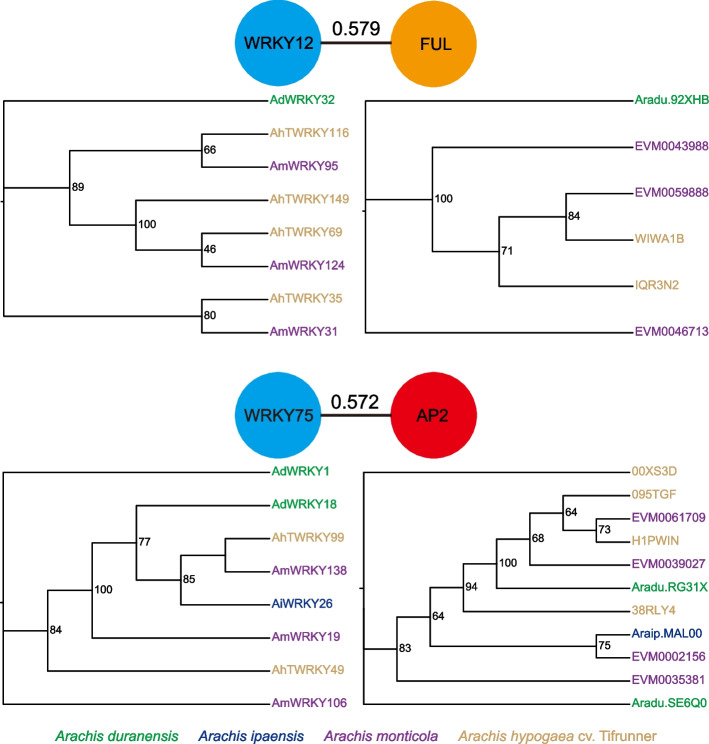
WRKY transcription factors interact with FUL and AP2 to modulate flowering time in the four *Arachis* species

The WRKY TF interaction relationships among the four *Arachis* species were predicted using the STRING database. The WRKY TFs, which were orthologs to AtWRKY12, interacted with FUL in the four *Arachis* species (Fig. 3). Similarly, the WRKY TFs, which were orthologs to AtWRKY75, interacted with APETALA2 (AP2) in the four *Arachis* species (Fig. 3). Phylogenetic analyses showed that WRKY TFs and the associated proteins exhibited different evolutionary relationships in the four *Arachis* species. FUL showed a high likelihood of loss in *A. hypogaea* cv. Tifrunner genome compared to the WRKY12 TFs (Fig. 3). Conversely, AP2 was prone to expansion relative to WRKY75 in *A. monticola* and *A. hypogaea* cv. Tifrunner genomes (Fig. 3). Notably, there was no evidence confirming that AtWRKY12 and AtWRKY75 proteins interact with FUL and AP2 to modulate flowering time. The results indicate that WRKY TFs exhibit specific protein interaction relationships to modulate flowering time in the four *Arachis* species.

### Varying expression patterns of flowering-time genes in 22 tissues of *A. hypogaea* cv. Tifrunner

**Fig. 4:**
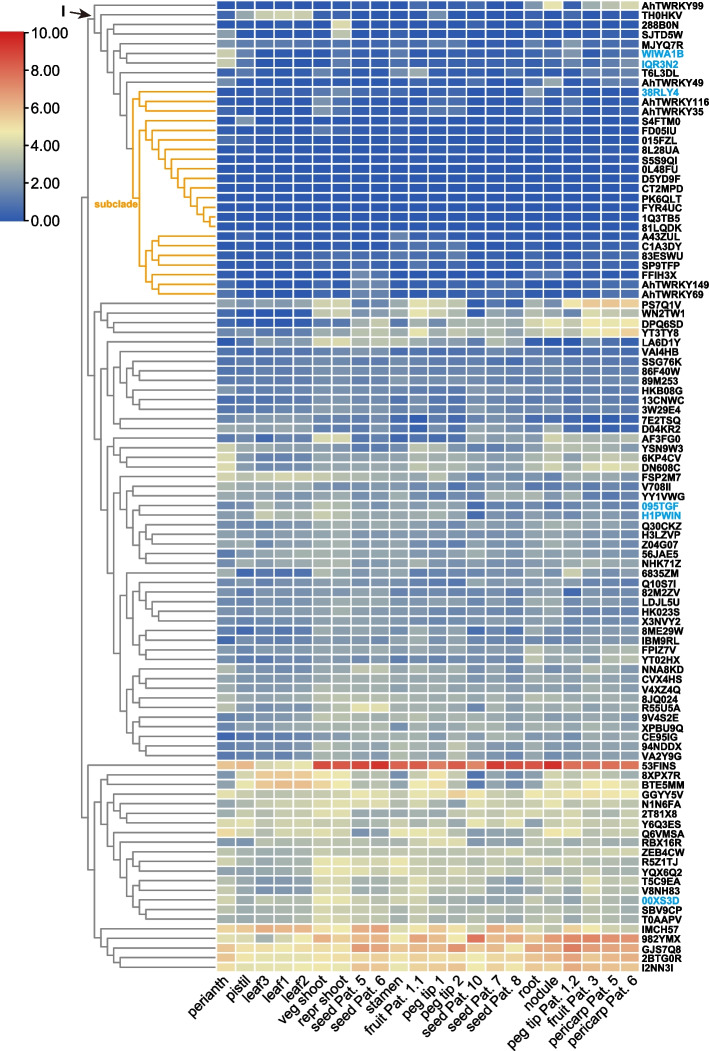
The expression patterns of WRKY and flowering-time genes in 22 tissues of *Arachis hypogaea* cv. Tifrunner. Blue font indicates interacting proteins with WRKY transcription factors

WRKY TFs regulating flowering time clustered in group I based on their expression levels in 22 tissues of *A. hypogaea* cv. Tifrunner (Fig. 4). The genes in group I exhibited low expression levels compared to other groups (Fig. 4). Group I had two homoeologous gene pairs, AhTWRKY35 and AhTWRKY116, and AhTWRKY69 and AhTWRKY149. Each homoeologous gene pair exhibited similar expression pattern in 22 tissues (Fig. 4). These results indicate that WRKY homoeologous gene pair share similar regulatory networks. In addition, 18 flowering-time genes were grouped in a subclade based two the WRKY homoeologous gene pairs (Fig. 4), indicating that these genes are potentially regulated by these WRKY homoeologs.

In addition, AhTWRKY49 and AhTWRKY99 (ortholog with WRKY75) indicated potential synergistic interactions with 38RLY4 (AP2), and AhTWRKY35 and AhTWRKY116, and AhTWRKY69 and AhTWRKY149 (ortholog with WRKY12) exhibited potential synergistic interactions with WIWA1B and IQR3N2 (FUL), because they had similar expression patterns (Figs. 3 and 4). Conversely, AhTWRKY49 and AhTWRKY99 exhibited potential antagonistic interactions with 00XS3D, 095TGF, and H1PWIN (AP2) due to the differences in expression patterns (Figs. 3 and 4).

## Discussion

*Arachis* plants are essential sources of oil, proteins, and forage [13]. The genomes of cultivated peanut and its progenitors have been sequenced [15, 17, 32–34]. WRKY TFs have been identified at the genome level in members of *Arachis* genus [5, 21]. However, the functions of WRKY TFs in flowering time have not been elucidated. In this study, WRKY TFs that regulate flowering-time genes and their interaction proteins in *A. duranensis*, *A. ipaensis*, *A. monticola*, and *A. hypogaea* cv. Tifrunner were identified using bioinformatics approaches. The main findings are summarized as follows: Firstly, WRKY TFs are involved in aging, autonomous, circadian clock, hormone, photoperiod, sugar, temperature, and vernalization pathways to modulate flowering time in the four *Arachis* species. Secondly, asymmetric WRKY TFBSs were identified in homologs of the flowering-time genes across the four *Arachis* species. Thirdly, two conserved protein complexes involving WRKY TFs interaction with FUL and AP2 modulated flowering time in the four *Arachis* species.

WRKY TFs are primarily involved in flowering pathways and phytohormone pathways to modulate flowering time [4, 35]. The flowering pathways include photoperiod, autonomous, vernalization, gibberellin, and aging pathways [4, 6]. WRKY TFs regulate flowering time through ABA, auxin, and ET pathways [4, 10, 36]. However, the role and the underlying mechanisms of WRKY TFs in regulating flowering time through sugar and temperature pathways have not been fully elucidated. Previous studies demonstrated that low sugar and high temperature promote flowering, whereas high sugar and low temperature conditions delay flowering in plants [4, 37–39]. These findings imply that WRKY TFs potentially modulate flowering time through sugar and temperature pathways in the four *Arachis* species.

Specific interactions between WRKY TFs and other proteins modulated flowering time in the four *Arachis* species. *Arachis* WRKY TFs interacted with FUL and AP2 proteins to modulate flowering time. FUL, a member of the MADS-box gene family, modulates flowering time and floral meristem development [40, 41]. AP2, a member of the AP2/ERF gene family, regulates the interaction between floral meristem and APETALA1 (AP1), LEAFY (LFY), and CAULIFLOWER (CAL) to modulate flower and floral meristem development [42, 43]. Notably, a regulatory network exists between FUL and AP2. FUL directly and negatively regulates AP2 to downregulate wuschel-related homeobox expression in shoot apical meristem, reducing flowering in monocarpic plants [44]. Furthermore, AP2 downregulates genes implicated in axillary bud dormancy and cytokinin signaling, resulting in global proliferative arrest to affect flowering in monocarpic plants [45]. These findings indicate that WRKY TFs are implicated in the global proliferative arrest pathway in *Arachis* genus.

*Arachis* WRKY TFs, which regulate flowering time, are orthologs with AtWRKY12 and AtWRKY75 [5]. AtWRKY12 and AtWRKY75 interact with DELLA proteins to modulate flowering time [8, 31]. Moreover, AtWRKY12 activates *FUL*, and AtWRKY75 activates *FT* to promote flowering [8, 31]. Based on these results, we hypothesized that *Arachis* WRKY TFs, orthologous to AtWRKY12, interact with FUL protein and regulate *FUL* expression.

However, our study had some limitations. Firstly, it is challenging to establish one-to-one regulatory relationships between WRKY TF and downstream genes using bioinformatics approaches. This is because WRKY TFs regulate a conserved W-box element shared across several downstream genes. Additionally, our results showed that the number of downstream genes was higher than the number of WRKY TFs in the four *Arachis* species. These findings indicate that one WRKY TF may regulate several downstream genes to modulate flowering time. AtWRKY63 activates *COOLAIR* and *COLDAIR*, leading to the downregulation of *FLOWERING LOCUS C* (*FLC*) expression and consequently accelerating flowering [46]. AtWRKY71 activates *FT* and *LFY* to promote flowering [7]. Our results demonstrated that WRKY TFBSs of homologous downstream genes exhibit asymmetric evolution, suggesting that WRKY TFs interact with other transcription factors to modulate the flowering process in the four *Arachis* species.

## Conclusion

In summary, bioinformatics approaches were used in this study to predict the WRKY TFs regulating downstream genes and interaction proteins in the four *Arachis* species. WRKY TFs are involved in multiple pathways to modulate flowering time in the four *Arachis* species. WRKY TFs can interact with FUL and AP2 modulated flowering time in the four *Arachis* species. Although several novel regulatory networks were elucidated in this study, further experimental testing is required to verify these relationships.

### Supplementary Information

Supplementary Material 1.

Supplementary Material 2.

## Data Availability

The datasets generated and/or analyzed during the current study are available in the public databases as followings. Flowering-time genes from PFGD database: http://pfgd.bio2db.com/index.html.

## Abbreviations

ABA: Abscisic acid
AP1: APETALA1
AP2: APETALA2
CAL: Cauliflower
FLC: Flowering locus C
FPKM: Fragments per kilobase of transcript per million mapped reads
FT: Flowering locus T
FUL: Fruitfull
LFY: Leafy
PFGD: Flowering-time gene database
TF: Transcription factor
TFBS: Transcription factor binding site
